# “I’m standing next to him, I’m supporting him”—Supporting a loved one with brain cancer to donate their brain: A qualitative study

**DOI:** 10.1093/nop/npae049

**Published:** 2024-05-30

**Authors:** Cassandra P Griffin, Melissa A Carlson, Marjorie M Walker, James Lynam, Christine L Paul

**Affiliations:** Mark Hughes Foundation Centre for Brain Cancer Research, University of Newcastle, Newcastle, New South Wales, Australia; Hunter Medical Research Institute, Newcastle, New South Wales, Australia; School of Public Health and Medicine, University of Newcastle, Newcastle, New South Wales, Australia; Mark Hughes Foundation Centre for Brain Cancer Research, University of Newcastle, Newcastle, New South Wales, Australia; Hunter Medical Research Institute, Newcastle, New South Wales, Australia; School of Public Health and Medicine, University of Newcastle, Newcastle, New South Wales, Australia; Hunter Medical Research Institute, Newcastle, New South Wales, Australia; School of Public Health and Medicine, University of Newcastle, Newcastle, New South Wales, Australia; Mark Hughes Foundation Centre for Brain Cancer Research, University of Newcastle, Newcastle, New South Wales, Australia; Hunter Medical Research Institute, Newcastle, New South Wales, Australia; School of Public Health and Medicine, University of Newcastle, Newcastle, New South Wales, Australia; Department of Medical Oncology, Calvary Mater, Newcastle, New South Wales, Australia; Mark Hughes Foundation Centre for Brain Cancer Research, University of Newcastle, Newcastle, New South Wales, Australia; Hunter Medical Research Institute, Newcastle, New South Wales, Australia; School of Public Health and Medicine, University of Newcastle, Newcastle, New South Wales, Australia

**Keywords:** biobanking, brain cancer, glioblastoma, palliative care, postmortem

## Abstract

**Background:**

Brain cancer is a devastating and incurable disease that places a high burden of care on next of kin (NOK). NOK can play a core role in supporting end-of-life planning, including the decision to donate one’s brain after death. Postmortem brain donation is crucial to research. As postmortem programs develop it is important to understand the experiences of NOK as they support a loved one in the donation decision.

**Methods:**

Thirteen qualitative interviews were completed with NOK of people who had consented to donate their brains to the Mark Hughes Foundation (MHF) Biobank. A thematic analysis was carried out on the transcribed interviews.

**Results:**

Four central themes were identified: (i) The carer role has additional responsibilities and psychological benefits when brain donation is being considered; (ii) Supporting a loved one to donate requires mutual trust, understanding, and a commitment to honor agency; (iii) Increasing awareness of brain donation is a priority for NOK, and (iv) Brain donation is seen as a natural continuation of the donor’s altruistic values.

**Conclusions:**

When a person with brain cancer decides to donate their brain to research, their NOK can experience additional burdens and benefits as the NOK–patient relationship evolves. Understanding this evolution and recognizing the importance of trust, advocacy, and altruism provides a guide for the integration of brain donation programs into clinical pathways and a basis for normalizing brain donation as an extension of organ donation frameworks.

Patients with grade IV brain tumors experience a debilitating and often rapidly terminal disease.^1^ The limited performance of existing medical interventions including surgical intervention, chemotherapy, and radiation therapy has resulted in a median overall survival of 14–18 months for those diagnosed with glioblastoma (GBM).^2^ The role of NOK caring for those with high-grade brain cancer varies to that of other diseases due to the functional and neurological deficits experienced by patients—often exacerbated by behavioral and personality changes.^1^ Debilitating symptoms include headaches, vision loss, seizures, speech disturbance, and paralysis. As the disease progresses, patients can become increasingly reliant on their NOK for support with activities of daily living.^1,3^

Following a brain tumor diagnosis, immediate family or close friends are often expected to take on caregiver roles with little, if any, preparation.^4^ As a result, caregivers are reported to experience a range of unmet supportive care needs leaving them at risk of emotional distress during this time^5^ due to social isolation, feeling misunderstood, and an inability to talk about their feelings or situations.^6^ As has been acknowledged by Linendoll et al. (2008),^7^ the multidimensional nature of the caregiver role for NOK in primary brain cancer makes an assessment of needs and the provision of support highly individualized. Therefore, studies relating to specific stages or processes in carer journeys are essential to understanding the ways in which adequate support can be provided to NOK.

For many with advanced primary brain cancer, care teams will provide opportunities to engage in research such as clinical trials or biobanking programs. The opportunity to donate tissue to a postmortem biobanking program requires substantial planning and organization which has been well characterized in the literature due to the number of brain banks established for neurodegenerative diseases such as the New York Brain Bank.^8^ Postmortem brain donation programs are becoming recognized as primary translational research infrastructure platforms that are central to improving the understanding of brain tumor neurobiology.^9^ While invaluable from a biomedical standpoint, it has long been recognized that postmortem brain donation programs across neuro-oncology, psychological disease, and neuro-degenerative disease research are subject to unique social, ethical, and psychosocial factors that require additional consideration compared to the donation of surgical samples or liquid tissues.^10^ There is a burgeoning literature base^11^ established to better understand the motivations and barriers of donors when considering a postmortem program with many studies focusing on diseases such as Alzheimer’s^12^ or Dementia^13^ or with respect to healthy aging cohorts.^14^ What continues to be absent from the literature, however, are investigations that consider the unique context of brain cancer and the experience of NOK caring for a loved one.

In both organ donation for research and organ donation for transplantation, NOK plays a critical role in ensuring donation proceeds. Internationally the policy and legislation concerning organ donation differs, however, in many countries, NOK retain the right to refuse the collection of tissues for organ donation or postmortem research, at any point following the circulatory or brain death of a loved one.^15^ Therefore, the success of brain donation programs is partly dependent on NOK and their willingness to support donors during the decision-making and consent processes.

While not specific to brain donation, in their work exploring perceptions of organ donation, Riley et al. identified that the ability of NOK to participate in organ donation decisions at the end of life could assist them to overcome feelings of powerlessness and simultaneously generate a sense of meaning from senseless tragedy.^16^ While obtained in an organ donation for transplantation setting, Riley et al.’s findings complement those of Eatough who identified that playing a critical role and ensuring donation went ahead was a welcome distraction for next of kin of brain donors, describing it as a “last act” that provided a sense of focus, control, relief and comfort.^17^ Providing particular relevance for NOK of those with end-stage brain cancer, Eatough’s work suggests that the control afforded by participating in brain donation eased the strain of relinquishing care after a protracted illness, proving a much-needed practical focus.

Obtaining a greater understanding of NOK–donor relationships, and the challenges faced by NOK is crucial for ensuring adequate support is provided. This study aims to qualitatively characterize the experiences of NOK carers who are supporting a loved one with brain cancer to consent for brain donation and to better understand their lived experience of the process. In doing so, brain banks can provide additional support and review pathways for future participants and NOK.

## Materials and Methods

Ethics approval was granted for this study by the Hunter New England Human Research Ethics Committee (HNEHREC 2018/ETH00261).

### Participants and Setting

Participants were recruited in conjunction with the Mark Hughes Foundation (MHF) Brain Cancer Biobank. Any NOK who was supporting an individual to donate to the MHF Brain Cancer Biobank was eligible to participate in the study.

### Procedure and Measures

Recruitment to the MHF Brain Biobank occurs largely via specialist brain cancer care coordinator (BCC) nurses or through the treating clinician. In many cases, patients will self-nominate to a clinical team member having read or heard about the biobank and wanting further information, or in others the HCP may raise the opportunity if the patient openly discusses a keen interest in research. Potential participants were invited to participate upon introduction to the biobank team. Semi-structured interviews employed a participant-centered approach, allowing each participant the chance to tell their story and the interviewer scope to follow up on points or statements relevant to individual journeys. The interview schedule focused on views and feelings throughout the consent process, their rationale and hopes for outcomes as well as concerns about potential logistical challenges. Interviews were conducted in person at the time of consent or via telephone after the consent of the donor was completed where preferred. These interviews were recorded and then transcribed. The interviews were conducted between (March 2021 and November 2023) by the biobanking and clinical research manager (CG) who is responsible for consenting potential brain donors and their NOK. Sampling was continued until it was determined that the sample of participants provided rich breadth and depth for analysis, rather than saturation for thematic analysis.^18^

### Interviewer Perspective

It is acknowledged that the interviewer (CG) established connections and rapport with many of the participants and in the majority of cases would be present at a later time during the donation itself. As such, it is recognized that the researcher is personally present in the study, is committed to the positive value of brain donation and contributes personal experience and insights to the interpretation of results in this phenomenological study.

### Analysis

Analysis was facilitated with the use of NVivo (Version 12 Pro, QSR International) and was conducted by 2 authors (C.G. and M.C.). All responses were coded and reflexively discussed following which a reflexive thematic analysis with narrative synthesis was conducted to explore the sequential organization of events and understand the components of each theme within the wider brain donation paradigm. Guided by the methodology of Braun and Clarke,^19^ data were coded based on keywords, phrasing, and expressed sentiments. These codes were then grouped based on parallels in language and contextual factors and used to construct themes which could be illustrated and presented in narrative form with the use of demonstrative quotes. It is recognized that both authors interpreted through a personal lens with one highly present in the data due to direct involvement in the consent and facilitation of donation and the other external to the program.

## Results

A total of 13 NOK supporting 11 donors consented to participate in the study. Table 1 details study participation and basic demographic data along with the relationship to the donor for each respondent. Occupational categories were informed by ISCO-08^20^ though not strictly followed to ensure heterogeneity was adequately captured Table 1.

**Table 1.** Participant Demographics

Demographic	Range/category	*N*
Age	20–30	1
	30–40	2
	40–50	1
	50–60	3
	60–70	3
	70–80	3
Occupation	Professional	8
	Skilled Trade	3
	Technician	1
	Not Documented	1
Relationship	Mother	1
	Father	2
	Sister	1
	Wife	5
	Husband	2
	Daughter	1
	Friend	1

Following a review of the transcripts along with coding and analysis of the data, 4 key themes were developed.

**Theme 1: “I’ve got to be up there right at the front”—the carer role has additional responsibilities and psychological benefit when brain donation is being considered.**

The data indicated that while the degree to which NOK was involved in end-of-life decisions varied, NOK believed it was their role to support the choices made by potential donors.

*NOK 1 “I think he’d already made it, and he just used me as a sounding board.”*

*NOK 2 “I just respected her decision… I’m not going to change her mind. It’s [her] wish, and she’s adamant about it, and passionate about it… my role is to get the process right when the big day comes.”*

*NOK 10 “I just said whatever you want to do…you’ve got to be comfortable with it, and I will support you in whatever you do.”*

Some NOK expressed caution regarding their role, referencing the need to share information without influencing their loved one’s decision.

*NOK 8 “I raised it, but I wouldn’t have made the decision…I don’t know if I would have any right to ask [him] to think about it…I raised it in just a general way... And then when I spoke about that, he made the decision… I would never have said, ‘is this something you’d like to do?’”*

NOK believed they played a key role in obtaining sufficient information for potential donors to make an informed decision.

*NOK 10 “[He] said to me, you need to ask the questions. How can I do this?”*

The sharing of information between donors and NOK further established the role of NOK as an active participant in the process, tasked with the responsibility of ensuring donation is carried out.

*NOK 7 “She told me… this is something that she wanted to do and here’s the information… When she does pass away, there’s actions that have to be made in a timely manner…It’s very important that the people closest to her are aware of her wishes so that they can be fulfilled.”*

NOK expressed that their responsibility to honor wishes is a crucial part of their role, integrated into the practical realities of being a carer to someone with terminal brain cancer.

*NOK 12 “For me, if I can do lots of preparation …getting the house done… getting his funeral organised, for me, it’s one more step in the planning.”*

*NOK 6 “The whole conversation is about after she’s gone.... [It’s prompted me to think] when to say goodbye, how to say goodbye, will she still be on a ventilator when I say goodbye?”*

Ultimately NOK felt the weight of responsibility associated with ensuring the donation was successful.

*NOK 11 “If something goes wrong in terms of getting it to you… that’s my biggest concern…will we be able to get everything rolling in that time?*

Despite the weight of responsibility, having a practical objective provided a source of focus and comfort, especially for carers looking for something “tangible” beyond daily care responsibilities.

*NOK 4 “About three months ago, I started to go into that, ah, okay, I can’t do anything…And that’s when [focusing on donation] became a comfort to me.”*

*NOK 5 “As a carer, you’re doing just about everything... There’s not a lot that you can do to change the situation, but this does make a difference to [them]…That’s what he wants and it’s not my place to judge it, … it’s my place to support him… I guess it makes me a bit stronger.”*

*NOK 4 “You’re kind of on auto, you’re doing all this meaningless shit. And then all of a sudden someone says you can make a difference…I was so drained and so tired.... After the phone call [pre consent], I just felt so much calmer and awake, and I kept saying to her, I’m okay, it is okay now.”*

Ensuring donation proceeds were of significant value to NOK, and failure to ensure donation may have a potentially devastating impact. This was demonstrated by 2 NOK who were initially told donation would not be possible due to the unconscious state of the donor and incomplete consent paperwork.

*NOK 3 “Bloody devastated. I had my little crying sessions…because I wasn’t going to be able to fulfil [her wish]. And that’s what it’s all about, just what she wants, and what she doesn’t want.”*

*NOK 4 “It’s something to cling onto, because initially they’d said it’s probably too late. And I was gutted, because all that I can do is try and follow out these little wishes. I felt immense comfort [after consent], I’ve walked around like a lunatic with a smile on my face.”*

**Theme 2: “I’m really glad that she made me enduring guardian, because we’ve had them, the hard discussions…that is her right and her wish”—supporting a loved one to donate requires mutual trust, understanding and a commitment to honour agency.**

Reciprocal trust underpinned the relationship between potential donors and NOK, with NOK trusting donors to be fully informed and make the decision that is right for them.

*NOK 1 “[I told him] just make sure you look into it all …he usually goes into a fair bit of detail on stuff beforehand, he does a lot of research.”*

In turn, there was an understanding that NOK would provide an honest and well-considered response when asked about their thoughts on brain donation by donors.

*“NOK 11 “she feels that she can talk to myself and my son about things, and we will give her an honest answer…I think that’s what she understands, that we don’t just go willy-nilly and say yes or no to her questions or her decisions.*

Given the nature of the disease and the potential for it to impact decisional capacity, NOK felt strongly that they must advocate for the views of their loved ones. There was a perceived onus of responsibility on NOK to encourage and respect the agency of potential donors, understanding that a time would come when they would be trusted to make decisions on a donor’s behalf.

*NOK 5 “It’s something that he’d decided himself…although it affects me as well, it’s very much his journey… He needs to be in control of the journey, and if that’s the journey he wants us to take, well that’s the journey we’ll take.”*

Once the decision had been made, NOK recognized that they were trusted by potential donors to ensure they honored their decisional agency and upheld their wishes.

*NOK 5 “I’ll be telling them in no uncertain terms this is his wish, and we are going to respect that whether we agree with it or not.”*

Overwhelmingly NOK expressed thanks and gratitude for the information that was provided to them by the health care teams.

*NOK 4 “I’m just so glad there’s people like you who are doing all the behind-the-scenes stuff. You’re giving her this indirect dignity and allowing her to contribute to something more and I’m really thankful.”*

*NOK 6 “…they’re very efficient with finding information and getting information and the help from the whole hospital system, and the local Cancer Centre and that.”*

NOK also expressed that brain donation conversations could provide an avenue for end-of-life care planning which could be often difficult to raise, and to envision their own life during and after the death of their loved one.

*NOK 5 “It gives me a more finite idea of what he wants towards the end…it has definitely prompted him to have that conversation, where I don’t know if he would have had that conversation as openly as he has.”*

**Theme 3: “I just don’t think it’s being spoken about, and they need to…to try to get that conversation going”-increasing awareness of brain donation for research is a priority for NOK**

The data indicated that some but not all NOK were aware that postmortem tissue donation for the purpose of research was available.

*NOK 4 “I was happy to be an organ donor, but I didn’t, I really wasn’t aware that you could donate for research.”*

*NOK 9 “Yes, I was aware… if there was any part of me that was any good to research or anything else, it’s no good to me after I’m dead.*

In several cases, awareness of brain donation was triggered by the search for connection with those who had a similar experience.

*NOK 4 “I had talked about it with my friend, and she spoke to me about how [her husband] had found a lot of peace in [consenting to donate] …After talking to [her], I went back and told [donor] about it, and she said, that’s what I want to do.”*

NOK indicated that the way you become aware of brain donation matters—shaping your initial response. Timing played a large role, with 1 donor suggesting that “generic information” such as a story on TV, rather than a direct conversation with a member of the medical team, made the decision more palatable.

*NOK 12 “You always hold out for hope…And when it gets to that stage, then you know there’s no hope left. And that’s probably why things on TV are a couple of steps removed, it gives you a chance to think about it without it being directed at you.”*

NOK felt called to raise awareness of the donation process and the disease, believing it was not openly spoken about. Several felt that it was their role to talk to their communities to increase awareness.

*NOK 1 “Just to go back to talk to women who are in my era, to see what those conversations would be, that could be a start then, for them to be discussing it with their extended families as well.”*

Some participants indicated that there were societal taboos around discussions of death and dying that prevented awareness.

*NOK 1 “It’s one of those things that society’s just not ready for yet, but it would be great if you had a bit more advertising that these things are available, so people get used to the idea... Look how long it’s taken, for just a normal donor to be on licences and things like that….and euthanasia too… I just don’t think it’s being spoken about, and they need to have some good PR, to try to get that conversation going.”*

Others inferred that healthcare teams are reluctant to raise awareness with patients until the terminal phase of the disease unless prompted by the patients themselves prior.

*NOK 12 “Maybe it could be a little better publicised, because I guess it’s a hard thing, isn’t it? The neurosurgeon only brought it up because we’re at the pointy end of the journey now, and [donor] raised it first.”*

NOK felt their loved one’s contribution would directly impact research and awareness and hope their example will encourage others to follow suit, increasing the impact of their donation and triggering a research legacy.

*NOK 2 “It might even jog their memory and think, well I’d like to do something like that… it probably makes other people think it’s a good thing to do. And if it can get one other person to do the same, if they’re in that same situation, I think it’s a positive.”*

One participant reflected on the impact his wife’s consent had on their young son, noting the flow-on for fundraising and awareness.

*NOK 13 “I hope it inspires him later on down the track for something bigger and better… Since we told him Mummy was going to die about a month ago, he wants to do stuff at school… fundraisers and things like that and having it donated to the Mark Hughes Foundation.”*

While largely hopeful, the data contained a tone of frustration, suggesting governing institutions have provided insufficient advocacy to raise awareness of brain cancer or donation. NOK believed that with greater awareness would come increased support for research and improved outcomes.

*NOK 13 “I just hope [this interview] gets to the appropriate people, and people that potentially can make the difference, or are willing to try and make the difference.”*

*NOK 13 I think some of these politicians and people in power, they’ve all got their own vested interests… and I’m not the only person that’s going through [brain cancer]. Hopefully, someone at that level can stop for a second and think for their own family. How would they feel or how would they be if it was to happen to them?*

**Theme 4: “Donating organs. It’s always been a family thing. If we can, we will”—brain donation is seen as a natural continuation/extension of the donor’s altruistic values.**

When asked about their feelings towards postmortem donations, NOK aligned their views with organ donation for transplantation.

*NOK 12 “Well, I’ve been an organ donor since I got my driver’s licence, whenever it first came in to go on your driver’s licence. So, obviously I was pro that idea.”*

Altruism was the overwhelming association for NOK when asked about their feelings toward organ donation—both for research and transplantation.

*NOK 6 “I’ve always been in favour of it…I just think there’s so many people go through so much pain and suffering… we can prevent a lot of it.”*

*NOK 11 “The main positives… helping research and looking to the future, that somewhere along the line, someone will crack the little bit of information that will help people with brain tumours, someone in the future.”*

Many NOK recounted that they were “unsurprised” by these altruistic intentions, as the donor’s decision aligned with the donor’s prior actions or the motivations of the NOK.

*Nok 12 “He’s always been a fairly altruistic person, and he’s also been a long-time organ donor, or would be organ donor. … He’s giving his motorbike to his best mate, who loved it. And it’s just one more thing that he’s doing, that he thinks will make people happy, and they will remember him.”*

*NOK 9 “I’ve always wanted to [donate]. I had two little brothers who died when they were young…water on the brain was one, and the other one was kidney failure or something. And I always wanted to donate in case I could help [others like] them.”*

Altruism aside, NOK reported that donors can hold concerns around the association between the brain and “self” and that these concerns of “wholeness” can also be felt by NOK.

*NOK 11 “She may not be happy to know that part of her was going to be missing when she was going on to her next life.”*

*NOK 12 “He decided maybe he wouldn’t do it, because he wouldn’t be himself without his memory…I then…just had a moment myself where I thought is that what I want to do, am I losing a part of him? …and, of course, I slapped myself around the face.”*

*NOK 13 “That was her dad, as he felt that he wanted the whole part of [her] to be buried or cremated, whatever… He wanted his daughter to fully be there.”*

The presence of these concerns varied within the data with others taking an opposing view and suggesting donation of all tissue was largely equivalent.

*NOK 1 “I just take it in my stride. A blood donor or a brain donor…”*

Reflecting the highly individualistic nature of the data, 1 revealed that she was not herself an organ donor.

*NOK 11 “When it first started… maybe 20 years or so ago, my thoughts were that if something happened to me and the doctors decided that I was suitable for an organ donation but I wasn’t ready… the doctors would do it anyway because I’d signed the form. And that’s probably the main thing that stopped me from doing it…and I haven’t done it since.”*

Despite her concerns, she then reiterated her support for her loved one, citing that contributing to something greater than herself would provide her with a legacy.

*NOK 11 “She’s giving something back to the world… She’s led a fairly insular life... I think that this is her opportunity to expand her life a little bit. I know that sounds funny, expanding her life in death, but that’s how I feel. She’s giving something back to the world.”*

## Discussion

Our study, which aimed to characterize the lived experience of supporting a loved one with brain cancer to consent to a postmortem brain donation program, indicated that NOK views themselves as active participants in the process. Many view this as an extension of their carer responsibility, believing their role includes the provision of information, serving as a sounding board and ensuring that the donation proceeds once their loved one has passed. This mirrors the insights of Eatough et al.^17^

The role of the carer and the reciprocal trust between donors and NOK are identified as key themes within the data. With respect to the trust instilled in NOK and their ever-increasing role in patient support, as potential donors deteriorate, many NOK hold concerns regarding their ability to “influence” the decisions of potential donors. This is a caution that can be complicated by the clinical presentation of the disease. Edvardsson and Ahlstrom (2007) identified a change in roles and relationships between NOK and people with brain cancer and a loss of reciprocity in relationships.^21^ Changes in behavior and personality as well as cognitive changes complicate the decision-making process with respect to brain donation or any other planning for individuals with terminal brain cancer. As is recognized by Lien and Rohde (2021), changes such as an apathetic affect, loss of initiative and empathy, indifference, selfishness, impaired emotional control, and tendencies toward childish behavior can greatly complicate end-of-life planning.^22^ This increases the burden of responsibility for NOK who are tasked with ensuring potential donors make appropriate decisions while supporting and advocating for agency and decisional capacity. This challenge is not unique to brain cancer. Boise et al. noted the challenges associated with cognitive decline, decision-making, and communication styles with relevance to brain donation in an Alzheimer’s population.^23^ This study suggested that early decision-making was key to preserving NOK–donor relationships and that in instances where donor cognitive decline was present, consensus decision-making was essential among family members to avoid dissent or additional distress between carers.

In line with consensus decision-making, appropriate advocacy for brain donation is heavily dependent on the relationship between both parties and awareness or previous discussions around end-of-life planning, death, and dying. Eatough et al. recognized this responsibility, noting that NOK supported donation because it was “in keeping with” their loved one’s character or previous behavior—a sentiment that is consistent with the data presented. Recognizing that donation was aligned with character, or in many cases aligned with previous views on organ donation, allows NOK to make an informed decision and confidently validate and support their loved ones during the consent process.^17^

Reflections on the nature of communication between potential donors and NOK were recurrently seen across all themes, varying in nature due to the transition of the NOK’s role. As theme 1 illustrates, NOK is initially a sounding board or support and then transition to a trusted advocate required to make decisions or act in a manner that honors the donor’s agency when the donor no longer can. These insights carry specific relevance for end-of-life planning and the handing over of autonomy from the donor to NOK, specifically referenced by 1 participant who noted that end-of-life conversations were instigated by the donation conversation—a catalyst she was grateful for. End-of-life planning and measures such as bequests, advanced directives the appointment of an Enduring Guardian are referenced multiple times within the data—with NOK repeatedly noting there were difficulties instigating these conversations despite being key to the establishment of trust. It is recognized within the literature that early and high-quality conversations around end-of-life care should occur as close to diagnosis as possible to enable comprehensive care, however, evidence suggests that in most cases these conversations are mishandled or mistimed.^24^ This is further supported by comments made within the data regarding awareness and the willingness of the community to openly discuss topics associated with a death such as euthanasia.^25^ Wideheim et al. (2002) cited the positive impact that early conversations around end-of-life arrangements, including funeral plans, can have on the psychosocial well-being of NOK.^26^ Given the difficulties cited above and that these conversations are often postponed, the value of brain donation as a catalyst for end-of-life conversations should not be undervalued from the perspective of NOK.

Reciprocal trust is a key element of theme 2, and while this presents in numerous forms through both theme 2 and theme 1 the most tangible demonstration is the trust that consented donors place in NOK to ensure donation proceeds—explicitly outlined in theme 2. Global policy regarding organ donation consent differs, with organ procurement organizations in the United States of America able to override NOK if donors are registered, while other nations favor explicit or presumed consent models.^27^ Rodrigue et al. (2008) described the “instability of organ donation decisions,” finding that more than one-third of NOK who do not consent to their loved one’s donation express regret at their decision.^28^ Of the nonconsenting NOK, 27% declined on the grounds that they had never held a conversation regarding donation with the deceased—despite being fully aware that the deceased was a registered organ donor. This subgroup later expressed regret at not honoring the deceased’s wishes.

Within the MHF biobank program, 100% of consented donors have proceeded to donate and it can be postulated that this is due to the frank discussions facilitated between potential donors and NOK during consent. The data here suggest that while the NOK of those who consent to brain donation are aware that refusal is within their rights, they largely hold the view that such a refusal would be disregarding their loved one’s wishes. The commitment of NOK and the trust built through these conversations empower NOK to take an active role in the donation process and minimize decisional instability.

### Limitations

This study was conducted within an Australian population. Due to the wide variability in international organ donation policy, the experiences of NOK documented here may not be transferrable. This study also did not interview individuals who chose not to proceed with donation. This has the potential to exclude or omit negative experiences. Therefore, studies of the experiences of those reluctant or who chose not to consent to brain donation are warranted, to provide a more complete understanding of the experiences of NOK during brain donation decision-making.

## Conclusion

This study indicates that NOK believe they play an integral role in the brain donation process, ranging from supporting decision-making, acquiring information and advocating for donation once the donor has passed. Reciprocal trust is paramount to both NOK and potential donors, and NOK who support loved ones to donate feel compelled to contribute to advocacy initiatives and to raise awareness of the impact of brain cancer—supporting the decisions of their loved ones through action. Conversations around donation can also provide a gateway to essential discussions around end-of-life planning that are otherwise difficult to catalyze in the short timeframe associated with a diagnosis of advanced brain cancer. This suggests that raising the topic of brain donation with patients and their loved ones early in the brain cancer journey and normalizing discussions of postmortem arrangements as an extension of organ donation paradigms, may have positive psychosocial implications for both patients and their NOK.
